# Risk factors and outcomes of early postoperative complications following congenital heart surgery: a retrospective study at a tertiary care hospital in Nairobi, Kenya

**DOI:** 10.1186/s13019-026-04403-3

**Published:** 2026-07-25

**Authors:** Nasra Ismail Adan, James Orwa, Ismail Ahmed, Mark Awori, Sean Del Rossi

**Affiliations:** 1 Department of Pediatric Cardiology, Aga Khan University, Nairobi, Kenya; 2 Department of Population Health, Aga Khan University, Nairobi, Kenya; 3 Department of Pediatric Cardiothoracic Surgery, Gertrude’s Children Hospital, Nairobi, Kenya

**Keywords:** Heart Defects, Congenital, Cardiac Surgical Procedures, Postoperative Complications, Oxygen Saturation, Intensive Care Units, Pediatric, Kenya

## Abstract

**Introduction:**

Congenital Heart Disease (CHD) remains a major health challenge globally, with surgical interventions being the primary treatment modality for many defects. While advancements in cardiac surgery have improved survival rates, early postoperative complications continue to pose risks, particularly in settings with limited resources like Kenya. The study aimed to assess the rate of any early postoperative complications and identify factors associated with it in children after cardiac surgery at a tertiary care hospital in Nairobi, Kenya from 2019 to 2024.

**Methods:**

This was an single-center retrospective study including children (< 18 years) who underwent cardiac surgery due to congenital heart disease at a tertiary care hospital in Nairobi, Kenya, over a 5-year period. Categorical variables were described as frequencies and percentages, while continuous variables were described as medians with interquartile ranges (IQR). Categorical variables were compared using chi-square or Fisher exact test and continuous variables compared using Mann-Whitney U test. Variables with p-value < 0.1 in the univariable analysis were included in the multivariable logistic regression, with p-value < 0.05 set as the level of significance. All analyses were performed using Rstudio version 4.5.1.

**Results:**

A total of 97 children were included in the study. The median age in months was 25.0 (IQR: 8.5–67.5) and 50 (51.5%) were male. Patent ductus arteriosus, ventricular septal defect, and atrial septal defect were the predominant preoperative diagnoses. At least one early postoperative complication occurred in 52.6% (95%CI: 42.6% to 62.5%) of the children. Preoperative oxygen saturation (AOR: 0.92; 95%CI: 0.85–0.98, *p* = 0.021) and closed heart surgery (AOR:0.32; 95%CI:0.11–0.86, *p* = 0.027) were significantly associated with early postoperative complications.

**Conclusion:**

The study findings highlight gaps and opportunities in perioperative care of children undergoing congenital heart surgery at a tertiary care center in Kenya. Preoperative oxygen saturation and closed heart surgery were independently associated with reduced odds of early postoperative complications. These findings suggest that optimizing preoperative physiological status and further investigating the factors driving the association between surgical approach and outcomes may inform strategies to improve perioperative care in this setting.

## Background

Congenital Heart Disease (CHD) is the commonest birth defect worldwide and constitutes a major global health problem [1]. CHD contributes substantially to the public health burden in developing countries, particularly in sub-Saharan Africa [2]. The prevalence of CHD varies globally and is approximately 8 per 1,000 live births [3]. Annually, about 1.5 million newborns are diagnosed with CHD, and over 4 million children around the globe are waiting for cardiac surgery [4]. According to the World Health Organization (WHO), Africa accounts for over 500,000 of the 1.5 million annual CHD cases [5]. In East Africa, Uganda estimates that 8300 children are born with CHD with nearly 2000 of them requiring cardiac intervention [6]. According to the population data and a report from the WHO, approximately 5,000 children in Kenya need congenital heart surgery annually. However, Kenya performs about 120–150 cardiac surgeries due to CHD, along with a comparable number of cardiac trans-catheter interventions [7].

CHD is the foremost cause of non-communicable disease-related deaths among individuals under 20 years of age [8]. With advances in medical technology and surgical techniques, the prognosis for children born with CHD has significantly improved in the past several years. Surgical intervention has become a crucial treatment modality, with operations performed to correct or palliate various cardiac defects. Despite the significant global reduction in mortality from CHD due to advancements in dedicated cardiac surgery centers and improved pediatric cardiac intensive care units, the situation in Africa remains dire.

Cardiac surgery is a high-risk procedure, and patients may develop any postoperative complications (POCs) involving both cardiac and extra-cardiac clinical systems. The Cardiac complications include arrhythmias, low cardiac output, need for reoperation and cardiac arrest. Extracardiac complications impact various systems, including the pulmonary, gastrointestinal, hepatic, neurological, endocrine, hematological, and renal systems. The inherent hemodynamic and physiological instability present in many children with CHDs may render these patients at high risk for adverse events [9]. The correction of most CHD generally requires complex surgical operations, catheter-based procedures, and specialized intensive care. Therefore, complications during these hospital admissions could considerably increase the mortality risk among infants and children. Nearly 33% of congenital heart surgeries results in either of the complications, thereby elevating the risk of death [9]. A study at Children’s Hospital Boston in Massachusetts revealed that 32% of pediatric patients encountered complications—78% of which were attributed to the procedure, 18% to devices, implants, or grafts, and 4% to medications [9]. A study conducted in Iran by Mirzaei and colleagues identified a notable rate of complications, with renal issues being the most frequent [10]. However, Javed et al. conducted a study in Jeddah, Saudi Arabia, focusing on infants younger than three months undergoing their first cardiac surgery for CHD. The most frequent complications observed were extended postoperative mechanical ventilation (27%), pleural effusion (21%), severe bleeding (19%), cardiac arrest (18%), and systemic infections [11, 18]. At tertiary hospital in Kenya, Tamar et al. examined the characteristics and surgical outcomes of cardiac patients with heart conditions present at birth or developed later in life in both adult and pediatric populations. They found that postoperative complications arose in 54.8% of pediatric cases, including acute renal failure (12.9%) and pneumothorax (9.7%) [12].

All open heart surgery (OHS) carry risks of complications related to CPB, such as insufficient organ perfusion, triggering of the systemic inflammatory response, embolism, and bleeding [13]. Cardiac surgery among children, especially infants, experiences a significant risk of morbidity and mortality. This is particularly pronounced when comparing surgeries performed with and without cardiopulmonary bypass [14]. A study conducted in Nashville, USA by Agarwal HS et al. showed that, factors linked to a higher incidence of cardiac complications in children undergoing CPB included prolonged CPB duration, elevated Risk Adjusted Classification for Congenital Heart Surgery (RACHS-1) scores, and reduced temperature during the CPB procedure [15]. Infants are particularly prone to noninfectious postoperative complications, including fluid accumulation and effusion, post pericardiotomy syndrome (PPS), capillary leak syndrome (CLS), and multiple organ failure [16]. Notably, PPS can affect up to 30% of infants undergoing cardiac surgery [17].

National benchmark data indicated that the overall mortality rate following cardiac surgery for CHD is below 4% at most institutions [15]. The mortality rate after infant congenital heart surgery differs considerably among centers, which is influenced by factors such as case complexity, additional comorbidities, and the specific type of heart surgery procedure performed [18]. Javed et al.,found that rates of in-patient mortality rates and one-month post-operative death rates were higher than those reported in two large studies conducted in the United States and United Kingdom, which were both under 5% [11].In Kenya the study by Tamara et al. reported a mortality rate of 6.5% among pediatric patients who underwent cardiac surgery [12].

Postoperative cardiac and extracardiac complications in pediatric cardiac surgery have been reported inconsistently, yet they play a significant role in mortality, length of hospital stay, healthcare costs, and overall quality of life following these procedures [10, 22].In a retrospective study, Agarwal et al. discovered that the post operative complications in pediatric cardiac surgery were linked to longer durations of mechanical ventilation, extended hospital stays, and higher mortality rates [15]. Understanding the types of complications and associated factors is essential for optimizing perioperative care, thereby reducing morbidity and mortality rates in post operated children [19]. To date, few studies have focused on postoperative complications and outcomes following congenital heart surgery in pediatric patients in Kenya. This study primarily aims to assess both cardiac and extracardiac complications during the early post-operative period and identify factors associated with postoperative complications in children after cardiac surgery at a tertiary care hospital in Nairobi, Kenya from 2019 to 2024.

## Methods

### Study settings

The study was conducted in the intensive care unit of Gertrude’s Children’s Hospital in Nairobi, Kenya. This non-profit facility is a 200-bed pediatric hospital with a 10-bed intensive care unit and a 5-bed high-dependency unit. Gertrude’s Children’s Hospital specializes in treating cardiac diseases, including congenital heart conditions, and performs approximately 30 open-heart surgeries annually in children with these conditions. The hospital operates a cardiac outpatient clinic six days a week, reviewing an average of 20 patients daily. Additionally, it has a well-established radiology department equipped with both cardiac computed tomography and magnetic resonance imaging scans for diagnosing congenital heart diseases. The cardiac unit is supported by a multidisciplinary team comprising pediatric intensivists, pediatric cardiologists, cardiothoracic surgeons, and pediatric intensive care nurses.

### Study design

This was a single-center retrospective study of pediatric patients who underwent congenital cardiac surgery between January 2019 and December 2024. The study employed a retrospective chart review methodology, analyzing existing medical records to extract relevant data on the study population.

### Study population and sampling

The study included all children who underwent congenital cardiac surgery during the study period at Gertrude’s Children’s Hospital in Nairobi, Kenya. Children were excluded if they underwent heart surgery due to acquired heart disease, if the surgery was performed at another facility other than the Gertrude’s Children’s Hospital, or if they were transferred to another facility within 24 h postoperatively. A formal sample size calculation was not performed due to the retrospective nature of the study, as all eligible children with complete information on the study variables were included.

### Data collection and management

Data for the study were obtained from medical records through an open chart review. A trained research assistant systematically reviewed the clinical records of all children who underwent congenital cardiac surgery. A standardized data collection form was used to extract relevant information from the records. The collected data were entered into REDCap, a secure web-based platform designed for building and managing online databases and surveys. Data extraction errors were minimized by having an independent reviewer checking 10% of the enter records for accuracy and completeness and also having an in-built logic within the electronic system to avoid saving missing data.

### Measurement of variables

The study collected data on demographic characteristics, medical history, preoperative risk factors, surgery details, postoperative complications, and postoperative management. Variables included age (years), weight (kg) at admission, gender, presence of preexisting medical conditions or clinical syndromes, preoperative diagnosis and type of surgery performed. Preoperative risk factors such as oxygen saturation, heart rate, temperature and laboratory data were also recorded.

Any early postoperative complication was defined as the occurrence of at least one complication in the pediatric cardiac intensive care unit (PCICU) during or immediately after surgery, up to hospital discharge. The complications were categorized by clinical systems: cardiovascular, neurological, pulmonary, gastrointestinal, renal, infectious, endocrine and hematologic. In addition, probable factors associated with complications included CPB support, CPB time, cross clamp time, heparin dosage, duration of mechanical ventilation, type or complexity of surgery were also collected.

### Statistical analysis

Continuous variables were presented as median values and interquartile ranges (IQR) and categorical variables as numbers and percentages. For univariable analysis, the Wilcoxon rank-sum test was used to compare continuous variables. The Pearson’s chi-square test or Fisher’s exact test were used to compare categorical variables and presence of the any early postoperative. Binary logistic regression analysis was performed to identify factors associated with any early POCs. A p-value < 0.05 was considered statistically significant throughout analyses. All data analyses were performed using RStudio version 4.5.1.

## Results

### Demographic characteristics

The study enrolled a total of 97 participants, with majority being male (51.5%, *n* = 50). The overall median age was 25 months (IQR: 8.5–67.5), and the median weight at admission was 11.0 kg (IQR: 6.5–19.5). The median gestational age in weeks was 39.0 (IQR: 38.0–39.0), and the median birthweight in grams was 3000 (IQR: 2600–3200). The median age at confirmed diagnosis of the heart condition was 6.0 months (IQR: 2.0–12.0).

There was a significant difference in children’s age (*p* = 0.015) and weight at admission (*p* = 0.003) with the lower values among those with any post-operative complications compared to those without any complications. The median length of stay in ICU and hospital in days were 3.0 (2.0–5.0) and 9.0 (7.0–13.0), respectively. Prolonged stays were observed among those with any postoperative complications than those without complication (Table 1).

**Table 1:** Demographic characteristics of children undergoing heart surgery at Gertrude’s children’s hospital, *N* = 97

-	-	Postoperative complications		
Characteristic	**Overall** *N* = 97	**No complication** *N* = 46	**Any complication** *N* = 51	*p*-value^1^
**Age (Months)**,** Median (IQR)**	25.0 (8.5–67.5)	39.0 (13.0–97.0)	14.0 (5.0–47.0)	0.014
**Gender**,** n (%)**				0.27
Male	50 (51.5)	21 (45.7)	29 (56.9)	
Female	47 (48.5)	25 (54.3)	22 (43.1)	
**Weight at admission (kgs)**,** Median (IQR)**	11.0 (6.5–19.5)	13.5 (8.4–22.5)	8.0 (5.6–15.0)	0.003
**Gestational Age at Birth**,** Median (IQR)**	39.0 (38.0–39.0)	39.0 (38.0–39.0)	39.0 (38.0–39.0)	0.82
**Birth Weight (gms)**,** Median (IQR)**	3,000.0 (2,600.0–3,250.0)	3,000.0 (2,500.0–3,100.0)	3,000.0 (2,800.0–3,400.0)	0.14
**Age at Confirmed Diagnosis**,** Median (IQR)**	6.0 (2.0–12.0)	7.0 (2.0–12.0)	5.5 (2.0–11.5)	0.58
**Duration of PCICU stay (days)**,** Median (IQR)**	3.0 (2.0–5.0)	2.0 (2.0–3.0)	4.0 (3.0–7.0)	< 0.001
**Duration of hospital stay (days)**,** Median (IQR)**	9.0 (7.0–13.0)	8.0 (6.0–10.0)	10.0 (8.0–15.0)	0.001

^1^Wilcoxon rank sum test; Pearson’s Chi-squared test

### Medical history

Pre-existing conditions and preoperative diagnoses are summarized in Table 2. Chromosomal abnormalities were present in 9.3% of children. Among these, chromosomal abnormalities, 77.8% (*n* = 7/9) had Down syndrome. The most common pre-operative cardiac diagnoses were patent ductus arteriosus (PDA, 43.3%), ventricular septal defect (VSD, 29.9%), and atrial septal defect (ASD, 20.0%). Less frequent preoperative diagnoses observed are detailed in Table 2.

**Table 2:** Medical history of children undergoing heart surgery at Gertrude’s children’s hospital, *N* = 97

Characteristic	*N* = 97
Chromosomal abnormality, n (%)	9 (9.3)
Other comorbidities, n (%)	26 (26.8)
Chromosomal abnormalities, n (%)	
Down syndrome	7 (77.8)
Others	2 (22.2)
Pre-operative diagnosis	
ASD, n (%)	20 (20.6)
VSD, n (%)	29 (29.9)
PDA, n (%)	42 (43.3)
AVSD partial, n (%)	2 (2.1)
AVSD intermediate, n (%)	1 (1.0)
AVSD Complete, n (%)	2 (2.1)
Aortic stenosis, n (%)	1 (1.0)
Pulmonary stenosis, n (%)	7 (7.2)
Coarctation of Aorta, n (%)	1 (1.0)
Tetralogy of Fallot, n (%)	9 (9.3)
d-Transposition of Great Atresia, n (%)	1 (1.0)
Tricuspid atresia, n (%)	7 (7.2)
Hypoplastic Heart Syndrome, n (%)	2 (2.1)

### Pre-operative risk factors

Preoperative clinical characteristics are summarized in Table 3. The median oxygen saturation was 96.0% (IQR: 91.0–99.0%), with significantly higher values observed in children who did not develop postoperative complications compared to those who did (*p* = 0.002).

Respiratory support was required in 17.5% (*n* = 17/97) of the children, comprising of nasal cannula (*n* = 10), non-invasive ventilation (*n* = 4), and intubation (*n* = 3). Cyanosis was present in 15.5% of children, and its prevalence of cyanosis was significantly higher among children who developed postoperative complications (23.5% vs. 6.5%; p-value = 0.021).

On preoperative medications, 33.0% (*n* = 32) of children received diuretics, 5.2% (*n* = 5) received inotropes, and 17.5% (*n* = 17) received other preoperative supportive medications.

**Table 3:** Pre-operative risk factors of children undergoing heart surgery at Gertrude’s children’s hospital, *N* = 97

Characteristic	Overall *N* = 97	No complication *N* = 46	Any complication *N* = 51	*p*-value^1^
**Preoperative oxygen saturation (%)**,** Median (IQR)**	96.0 (91.0–99.0)	98.0 (93.0–100.0)	95.0 (83.0–97.0)	0.002
**Preoperative respiratory support**				
Nasal Cannula, n (%)	10 (10.3)	1 (2.2)	9 (17.6)	0.017
Non-invasive ventilation, n (%)	4 (4.1)	1 (2.2)	3 (5.9)	0.62
Intubation, n (%)	3 (3.1)	0 (0.0)	3 (5.9)	0.24
**Presence of Cyanosis**,** n (%)**	15 (15.5)	3 (6.5)	12 (23.5)	0.021
**Preoperative medication**				
Diuretics (e.g. furosemide, spironolactone, hydrochlorothiazide, etc.), n (%)	32 (33.0)	12 (26.1)	20 (39.2)	0.17
Inotropes (e.g. epinephrine/adrenaline, norepinephrine/noradrenaline, milrinole, dobutamine, dopamine, etc.), n (%)	5 (5.2)	1 (2.2)	4 (7.8)	0.37
Other, n (%)	17 (17.5)	5 (10.9)	12 (23.5)	0.10

### Surgery details

Surgical characteristics and their association with postoperative complications are summarized in Table 4. Cardiopulmonary bypass (CPB) was employed in 67.0% of cases, with no significant difference in proportions in proportion between children who developed complications (74.5%) and those who did not (58.7%, *p* = 0.10). The median CPB duration in minutes was significantly longer in children who developed complication (121.0 min, IQR: 95.0-177.0) compared to those without complications vs. 69.0 min, IQR: 48.0–95.0; *p* < 0.001).

Among children who developed complications, 74.5% underwent open heart surgery compared with 25.5% who underwent closed heart surgery. In contrast, among those without complications, 54.3% underwent open heart surgery and 45.7% underwent closed heart surgery. Ventricular septal defect (VSD) repair was the most frequent procedure (23.7%), followed by atrial septal defect (ASD) repair (13.4%). Among the nine children undergoing tetralogy of Fallot repair, seven developed postoperative complications (77.8%).

The overall median surgery duration was 82.0 h (IQR: 4.0-240.0), with significantly prolonged procedures observed in children who experienced complications (*p* < 0.001). Median sternotomy was the predominant surgical approach (69.1%), whereas left sternotomy was performed in 30.9% of the cases (p-value = 0.036).

Core temperature during surgery remained consistent across groups (median 32.0 °C, IQR: 32.0–35.3 °C; *p* = 0.33). Intraoperative complications included bleeding and arrhythmias (four cases each). The other two intraoperative complications were isolated instances of atelectasis and cardiopulmonary compromise.

**Table 4:** Surgery details of children undergoing heart surgery at Gertrude’s children’s hospital, *N* = 97

-	-	Postoperative complications		-
Characteristic	Overall *N* = 97	No complication *N* = 46	Any complication *N* = 51	*p*-value^1^
**Cardiopulmonary Bypass (CPB)**,** n (%)**	65 (67.0)	27 (58.7)	38 (74.5)	0.10
**Duration of CPB (minutes)**,** Median (IQR)**	104.0 (64.0–129.0)	69.0 (48.0–95.0)	121.0 (95.0–177.0)	< 0.001
**Operation type**,** n (%)**				0.038
Open heart surgery	63 (64.9)	25 (54.3)	38 (74.5)	
Closed heart surgery	33 (35.1)	20 (45.7)	13 (25.5)	
**Types of surgical procedure**				
ASD repair, n (%)	13 (13.4)	11 (23.9)	2 (3.9)	0.004
VSD repair, n (%)	23 (23.7)	8 (17.4)	15 (29.4)	
TOF, n (%)	9 (9.3)	2 (4.3)	7 (13.7)	0.16
AV Canal Defect/AVSD repair, n (%)	5 (5.2)	2 (4.3)	3 (5.9)	> 0.99
PA (pulmonary Artery) Banding, n (%)	3 (3.1)	1 (2.2)	2 (3.9)	> 0.99
BT shunt, n (%)	2 (2.1)	0 (0.0)	2 (3.9)	0.5
Bidirectional Glenn Anastomosis, n (%)	4 (4.1)	1 (2.2)	3 (5.9)	0.62
Total Cavo-pulmonary connection/modified Fontan, n (%)	1 (1.0)	1 (2.2)	0 (0.0)	0.47
PDA Ligation, n (%)	38 (39.2)	21 (45.7)	17 (33.3)	0.21
Coarctation repair, n (%)	1 (1.0)	0 (0.0)	1 (2.0)	> 0.99
Other, n (%)	16 (16.5)	5 (10.9)	11 (21.6)	0.16
**Surgical approach**				0.036
Median Sternotomy, n (%)	67 (69.1)	27 (58.7)	40 (78.4)	
Left Sternotomy, n (%)	30 (30.9)	19 (41.3)	11 (21.6)	
**Duration of surgery (hours)**,** Median (IQR)**	82.0 (4.0–240.0)	4.0 (2.5–135.0)	194.5 (14.5–270.0)	< 0.001
**Cardiac arrest surgery**,** n (%)**	1 (1.0)	0 (0.0)	1 (2.0)	> 0.99
**Core temperature during surgery (degrees Celsius)**,** Median (IQR)**	32.0 (32.0–35.3)	32.0 (32.0–35.0)	32.0 (32.0–36.2)	0.38
**Intraoperative complications**				
Bleeding, n (%)	4 (4.1)	1 (2.2)	3 (5.9)	0.62
Arrhythmias, n (%)	4 (4.1)	2 (4.3)	2 (3.9)	> 0.99
Others, n (%)	2 (2.1)	0 (0.0)	2 (3.9)	0.5

^1^Pearson’s Chi-squared test; Wilcoxon rank sum test; Fisher’s exact test

### Postoperative complications

Postoperative complications are categorized by organ systems are shown in Table 5. The most frequent complications included coagulopathy in hematological disorders (*n* = 11), hypotension or hypertension in cardiovascular conditions (*n* = 14), acute kidney injury (AKI) in renal complications (*n* = 5), and mechanical ventilation not exceeding seven days in respiratory complications (*n* = 10). Additional complications observed were neurological deficits, mortality, sepsis, suspected infection, liver function test abnormalities, and ascites, electrolyte and metabolic disorders.

**Table 5:** Postoperative complications

Complications	*N* = 97
**Respiratory complications**	
Pneumonia, n (%)	1 (1.0)
Pulmonary Edena, n (%)	2 (2.1)
Pneumothorax, n (%)	3 (3.1)
Pleural effusion, n (%)	2 (2.1)
Mechanical ventilation (≤ 7 days), n (%)	10 (10.3)
Prolonged mechanical ventilation (> 7 days), n (%)	2 (2.1)
Other, n (%)	6 (6.2)
**Other post-operative complications**,** n (%)**	
Acute lung injury	1 (16.7)
Died	1 (16.7)
Emphysema	2 (33.3)
Lung injury	1 (16.7)
Stridor	1 (16.7)
**Cardiovascular complications**	
Low cardiac output/shock, n (%)	2 (2.1)
Arrythmias, n (%)	5 (5.2)
Hypotension/hypertension, n (%)	14 (14.4)
Use of temporary pacemaker, n (%)	5 (5.2)
Cardiac arrest/cardiopulmonary resuscitation, n (%)	2 (2.1)
Reoperation, n (%)	2 (2.1)
Other, n (%)	2 (2.1)
**Types of arrythmias complications**	
Tachyarrhythmias, n (%)	3 (3.1)
Bradyarrhythmia, n (%)	1 (1.0)
Other, n (%)	1 (1.0)
**Renal complications**	
Acute Kidney Injury (AKI), n (%)	5 (5.2)
Acute kidney injury not requiring renal replacement therapy, n (%)	1 (1.0)
Peritoneal dialysis catheter placement, n (%)	4 (4.1)
Fluid Overload, n (%)	2 (2.1)
Hematuria, n (%)	1 (1.0)
Other, n (%)	1 (1.0)
**Blood disorders**	
Bleeding requires chest exploration, n (%)	1 (1.0)
Bleeding requires transfusion, n (%)	5 (5.2)
Coagulation profile derangements (Prolonged PT and PTT time), n (%)	11 (11.3)
Other, n (%)	2 (2.1)

### Factors associated with the post-operative complications

In the univariable analysis, duration in PCICU (days), duration of hospital stay (days), preoperative oxygen saturation (%), weight at admission (kg), cross-clamp time (minutes), and CPB duration (minutes) were significantly associated with postoperative complications. After controlling preoperative and intraoperative variables in the multivariable model, preoperative oxygen saturation (%) and type of operation were significantly associated with postoperative complications. The likelihood of developing postoperative complications decreased by 8% for each one-percentage-point increase in preoperative oxygen saturation (AOR: 0.92; 95% CI: 0.85–0.97; p-value = 0.021). Similarly, the odds of developing postoperative complications were 68% lower among children who underwent closed heart surgery compared with those who underwent open heart surgery (AOR: 0.32; 95% CI: 0.11–0.86; p-value = 0.027) (Table 6).

**Table 6:** Factors associated with the post-operative complications among children undergoing heart surgery at Gertrudes children’s home

-	-	Univariable		Multivariable	
Variable	Type/category	OR (95%CI)	*p*-value	OR (95%CI)	*p*-value
Age in months	Continuous	0.99 (0.98-1.00)	0.051	1.00 (0.98–1.02)	0.786
Gender	Male		-		-
	Female	0.64 (0.28–1.42)	0.271		-
Duration in PCICU (days)	Continuous	1.85 (1.40–2.63)	< 0.001		-
Duration of hospital (days)	Continuous	1.17 (1.07–1.31)	0.004		-
Preoperative oxygen saturation (%)	Continuous	0.92 (0.85–0.97)	0.005	0.92 (0.85–0.97)	0.016
Operation type	Open heart surgery		-		-
	Closed heart surgery	0.43 (0.18-1.00)	0.053	0.32 (0.11–0.86)	0.027
weight at admission (kgs)	Continuous	0.95 (0.91–0.99)	0.027	0.97 (0.88–1.06)	0.537
Cross clamp time (minutes)	Continuous	1.03 (1.01–1.05)	0.014		-
CPB duration (minutes)	Continuous	1.03 (1.02–1.05)	< 0.001		-
Diuretics given	No		-		-
	Yes	1.83 (0.78–4.43)	0.172		-

## Discussion

To our knowledge, this is the first comprehensive description of early postoperative complications following pediatric congenital heart surgery at a private tertiary referral center in Kenya. The study included 97 children (median age 1.5 years, IQR 0.0–5.0) undergoing a spectrum of corrective and palliative procedures. Patent ductus arteriosus (41%), ventricular septal defect (31%), and atrial septal defect (22%) were the most common preoperative diagnoses. This pattern of predominant defects aligns with from studies in Uganda [20] and Tanzania [21]. The predominance of these defects across the region may be explained by factors such as delayed diagnosis and constrained access to early cardiac intervention [22], although preterm births have also been reported as contributing factors in the literature. In contrast, the median gestational age in our study population was 39 weeks, which is not in the preterm range.

In contrast, data from these regional studies generally reports VSD as the most frequent congenital heart defect, followed by ASD, with PDA representing a smaller proportion of surgical cases. The relatively high burden of PDA burden in our cohort which has also been reported in Ethiopia [23] likely reflects later presentation, referral patterns, and limited availability of catheter-based closure for infants in our setting. These differences highlight regional variations in diagnostic timing, resource capacity, and accessibility of specialized cardiac services when compared with high-income settings.

In this study, 53% of children experienced at least one early postoperative complication rate following heart surgery. This proportion is slightly lower than the 54.8% complication rate reported in a study in Kenya [24], however, it exceeds rates reported in several pediatric cardiac surgery series, including data from the Society of Thoracic Surgeons (STS) Congenital Heart Surgery Database (40.6%) [19], Tehran University of Medical Sciences in Iran with 26.7% complication rates [25] and, the national referral hospital in Indonesia at 19%. Similar program in sub-Saharan African, such as Ethiopia have also reported lower rates than the current study (39.5% [26]). Generally, early postoperative complication rates typically range between 20 and 40%, depending on case definitions, patient characteristics, and duration of follow-up. Nonetheless, direct comparison with our study findings remains challenging due to differences in case-mix, severity of the defects at presentation, and institutional resource capacity.

Several factors may have contributed to the higher complication rate observed in our study cohort. First, our broad definition of “any complication” likely increased inclusion relative to other studies that report only major postoperative morbidity. Second, resource limitations common in low- and middle-income settings—including constrained ICU staffing, limited access to advanced monitoring, and a high prevalence of preoperative malnutrition—have been consistently identified in Kenyan and broader sub-Saharan Africa literature as key determinants of early postoperative outcomes.

In our cohort, preoperative oxygen saturation and closed heart surgery were significantly associated with any postoperative complications. The common cause of hypoxia in the immediate postoperative period may occur due to residual effect of anesthetics, inadequate reversal, respiratory depression, airway obstruction and laryngospasm [27]. Higher preoperative oxygen saturation was associated with a reduced risk of any postoperative complications. The finding was consistent with the study linking perioperative hypoxemia with increased pulmonary complications after cardiopulmonary bypass in children [28]. Low preoperative oxygen saturation has been linked with the prolonged ICU stay in specific congenital heart disease populations, giving further emphasis on the importance of adequate oxygenation prior to cardiac surgery [29]. Studies further linking hypoxemia with adverse surgical outcomes in neonates found that lower preoperative oxygen saturation below 85% was independently associated with postoperative infection and extended ICU stay [30]. Prolonged ICU stay often an indication of complication severity in patients. Our study associated closed heart surgery with lower likelihood of getting any postoperative complications. Closed heart surgery is associated with shorter hospital stay, minimal blood loss and early mobilization with the closed heart surgery approach [31]. The current study findings is similar to that found fewer major complications among patients who underwent closed heard surgery compared with open heart surgery(OR: 0.52; 95% CI: 0.30–0.91) [32].

### Limitations

The study presented with some limitations. First, it was a single-center, retrospective study, which limits the generalizability of the findings to other private and public health facilities managing congenital heart conditions. Second, the study was based on routine data collected by the hospital which did not include many perioperative variables that may have influence on the outcome. Third, the sample size was relatively small and determined by the number of potential risk factors, thus limiting the power of the study to detect any possible associations. Finally, the study could not assess longitudinal trends in outcomes among the children.

## Conclusion

The study found Patent ductus arteriosus, ventricular septal defect, and atrial septal defect as the predominant defects in the preoperative diagnoses of the children. There was high burden of early postoperative complications in this setting which was associated with preoperative oxygen saturation and duration of ICU stay. The study findings highlight the critical gaps and opportunities in the perioperative care of children undergoing congenital heart surgery at a tertiary clinic in Kenya. Strengthening preoperative care with a focus on improving pre-operative oxygenation status together with postoperative monitoring and ICU capacity may help reduce the early postoperative complication rates. The study provides a foundation for larger multicenter studies within sub-Saharan Africa to better characterize risk factors, evaluate context-appropriate interventions, and develop predictive tools tailored to resource-limited settings.

## Data Availability

The datasets used and analyzed in this study are available from the corresponding author upon reasonable request.

## Abbreviations

CHDs: Congenital Heart Diseases
CLS: Capillary Leak Syndrome
CPB: Cardiopulmonary Bypass
HICs: High-income Countries
NCDs: Non Communicable Disease
OHS: Open-heart Surgeries
PCICU: Pediatric Cardiac Intensive Care Unit
POLOS: Postoperative Length Of Stay
POMV: Postoperative Mechanical Ventilation
PPS: Post Pericardiotomy Syndrome
RACHS-1: Risk Adjusted Classification for Congenital Heart Surgery, version
WHO: World Health Organization
